# Epigenetic (de)regulation of adult hippocampal neurogenesis: implications for depression

**DOI:** 10.1186/1868-7083-3-5

**Published:** 2011-11-01

**Authors:** António Mateus-Pinheiro, Luísa Pinto, Nuno Sousa

**Affiliations:** 1 Life and Health Sciences Research Institute, School of Health Sciences, University of Minho, Campus de Gualtar 4710-057 Braga, Portugal; 2 ICVS/3B's - PT Government Associate Laboratory, Braga/Guimarães, Portugal

**Keywords:** adult neurogenesis, depression, epigenetics, antidepressants, hippocampus, dentate gyrus

## Abstract

Adult neurogenesis represents a dynamic level of modulation upon the neuroplastic properties of the mature nervous system, that is essential to the homeostatic brain function. The adult neurogenic process comprises several sequential steps, all of which subjected to an assortment of cell-intrinsic and neurogenic-niche complex regulatory mechanisms. Among these, epigenetic regulation is now emerging as a crucial regulator of several neurogenesis steps. In particular, the active regulation of hippocampal neurogenesis and its repercussions in global hippocampal function are of special interest for the biomedical field, since imbalances at this level have been strongly related to the precipitation of several neuropsychyatric disorders, such as depression. Indeed, growing evidence supports that the detrimental effects on adult hippocampal neurogenesis, that have been associated with depression, might be epigenetically-mediated. Therefore, understanding the epigenetic regulation of the neurogenic process may provide a link between neurogenesis imbalances and the deterioration of the behavioural and cognitive domains frequently affected in depression, thus contributing to unravel the complex pathophysiology of this disorder.

Here, we outline some of the major epigenetic mechanisms contributing to the regulation of hippocampal neurogenesis and discuss several lines of evidence supporting their involvement on the development of imbalances in the neurogenic process, often correlated to behavioural and cognitive deficits commonly observed in major depressive disorder.

## Adult neurogenesis: the neurogenic process and its epigenetic regulation

### Neurogenesis in the adult brain

The beauty of research is that it ultimately defeats all established dogmas, even though some take very long to fall. Cajal's decree concerning the immutability of the central nervous system (CNS) has been reviewed and updated during the last decades, due to mounting evidence that substantiates the regenerative potential and plasticity of the CNS. Despite the initial reluctance manifested towards the first reports of post-natal neurogenesis, it is now well established that neurogenesis, a process that comprises the generation, differentiation and integration of new neurons in the preexisting brain neuronal networks, occurs in the adult brain, prevailing throughout life in specific brain areas, where neurons are persistently generated [1,2]. Such spatially defined brain regions where neurogenesis occurs display a permissive microenvironment for the maintenance and differentiation of neural stem cells and to their proliferation. Currently, two neurogenic brain regions are broadly recognized in the mammalian adult brain: the subgranular zone (SGZ) of the hippocampal dentate gyrus (DG) and the subependymal zone (SEZ) in the lateral ventricles.

In the hippocampal formation, the precursor cell population resides throughout the SGZ, with specific gradients [3]. After being generated in the SGZ, newly-born cells become committed to a neuronal lineage and migrate into the granule cell layer (GCL), where they mature to become excitatory glutamatergic granule neurons [4,5]. In the SEZ the precursor cells are mostly found in the anterior segment of the walls of the lateral ventricles. Here, newly-born precursor cells generate neuroblasts that will migrate along the rostral migratory stream (RMS), reaching the olfactory bulb (OB), where they fully differentiate mostly into granule inhibitory interneurons [6,7]. In addition to these two consensually accepted neurogenic regions, some authors have presented evidence that neurogenesis occurs in other brain areas, including the striatum [8], the cortex [9,10], the amygdala [11] and the hypothalamus [12,13]; however, as these results are still disputable [14,15], further studies are needed in order to elucidate if other neurogenic niches are indeed present in the adult brain.

Although it is now indisputably accepted that neurogenesis occurs in the adult brain, its functional relevance remains to be fully established. While it is clear that this phenomenon is confined to very discrete brain regions, the generation of new neurons in the post-natal period constitutes a new dimension of plasticity, with both direct and indirect impact on neuronal remodelling and repair, that is now regarded by the biomedical field as a promising therapeutical target in several neuropathological contexts. Notably, abnormal alterations in the hippocampal neurogenesis process have been implicated in an assortment of neuropsychiatric disorders [16-18]. Several research works, seeking to unveil the biological mechanisms underlying these disorders, became comprehensive studies about the hippocampal neurogenic process. Quite surprisingly, the functional relevance of adult neurogenesis in the SEZ has not yet been directly related to any specific neuropathological condition.

### A brief overview on adult hippocampal neurogenesis

**Figure 1:**
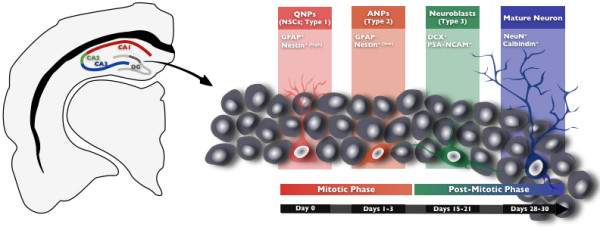
**Neurogenesis in the dentate gyrus (DG) of the adult rodent hippocampus**. The adult neurogenic process encompasses several highly regulated sequential steps. The process begins with the asymmetrical division of neural stem cells (NSCs), also named quiescent neural progenitors (QNPs or type 1 progenitors), giving rise to amplifying neural progenitors (ANPs or type 2 progenitors). ANPs start to exhibit the first signs of cell-lineage commitment and eventually exit the mitotic phase to become neuroblasts (type 3 progenitors). The neuroblasts will then differentiate and migrate towards its final destination where they will fully maturate into granular neurons and establish synapses within pre-existing circuits. Each cell stage can be distinctively identifiable by cell markers, some of which are indicated. It is currently assumed that the entire process of adult neurogenesis takes around 4 to 5 weeks. (GFAP - Glial fibrillary acidic protein; DCX **- **Doublecortin; PSA-NCAM - Polysialylated-neural cell adhesion molecule: NeuN - Neuronal Nuclei)

Integration of newly-born hippocampal neurons into pre-established neural networks seems to be achieved through highly regulated sequential steps: proliferation of neural stem cells, generation of amplifying progenitors, cell migration and, finally, maturation in the definitive destination with axon and dendrites formation and establishment of new synapses with preexisting surrounding cells [19-21] (Figure 1). This process of post-natal neurogenesis largely recapitulates the embryonic one, with the major difference that new neurons have to undergo these steps in an already mature microenvironment, having to integrate preexisting neural circuits.

The adult hippocampus SGZ contains an heterogeneous precursor cell population, distinctly identifiable through a particular set of molecules that each cell type expresses. The quiescent neural progenitors (QNPs) are believed to be the multipotent stem cells residing on the hippocampus [5,22]; they are also known as neural stem cells (NSCs) or, according to an alternative nomenclature, type-1 progenitor cells. Having both morphological and antigenic glial properties [23,24], they can be further distinguishable into two classes according to their spatial orientation: horizontal astrocytes (hA) and radial astrocytes (rA). These cells divide asymmetrically giving rise to daughter cells known as transiently amplifying neural progenitors (ANPs; also generally designated as type-2 progenitor cells). This phase of the neurogenic process comprises the emergence of the first indications of neuronal or non-neuronal lineage commitment [21], being for such reason, a decisive checkpoint in the determination of neural progenitors cell-fate. Anomalous alterations in this phase of the neurogenic process often result in long-term neuropathological traits [25]. Studies have showed that ANPs are highly mitotic [1,25], dividing symmetrically and giving rise to neuroblasts (NBs; also named type-3 progenitor cells). Neuroblasts are intermediate precursors in the generation of new granule neurons, expressing the microtubule associated protein doublecortin (DCX) that will be crucial for further maturation and migration [19,26]. Once the newborn cell becomes a neuroblast, it exits the proliferation cycle, and migrates towards its final destination in the GCL. Here, the newly-born cells will fully maturate, elongating their axons and establishing new functional connections, eventually becoming a mature granule neuron. The time window that takes to a newborn cell to be fully mature and integrated in the preexisting neural network is typically referred to be approximately five weeks [27,28]; however, some authors claim that the entire period of adult neurogenesis can take as much as 7 weeks [29,30], as this is the time required by the new neurons to become electrophysiologically indistinguishable from the remaining neuronal population.

Importantly, neurogenesis is a fine tuned process, rather than a mass phenomenon, during which most newborn cells are eliminated [31,32]. The mechanisms that regulate this neurogenic process are still to be fully understood, but recently, several studies proposed a complex epigenetic orchestration of adult hippocampal neurogenesis.

### Epigenetic orchestration of adult neurogenesis

**Figure 2:**
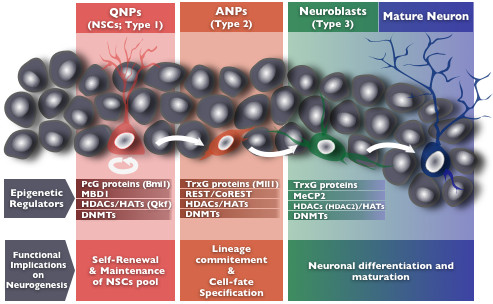
**Epigenetic regulators of the adult hippocampal neurogenic process**. The adult hippocampal neurogenic process is subjected to a complex epigenetic regulation, with important functional implications. Different types of epigenetic regulators have been identified, including PcG and TrxG protein complexes, MBDI, the REST/CoREST complex, MeCP2, HDACs, HATs and DNMTs, specifically involved in the fine tunning of the proliferation and specification of neural progenitors, as in the differentiation and maturation of the newborn neurons. Epigenetic regulators, such as the PcG protein Bmi1 and the methyl-binding protein MBD1 are involved in the regulation of the initial steps of neurogenesis, participating in NSCs self-renewal and maintenance. Later on the neurogenic process, the transcriptional activation of specific gene batteries by TrxG proteins like Mll1, together with the action of chromatin remodeling complexes such as the REST/CoREST complex and its molecular partners will allow the progenitor cells to exit the proliferation cycle and become committed to a neural cell lineage. Finally, the action of regulators such as MeCP2, will contribute to post-mitotic neuronal differentiation and maturation. Some epigenetic regulators, such as HDACs, HATs and DNMTs are involved in several regulatory checkpoints of the adult neurogenic process, integrating several protein regulatory complexes involved in the transcriptional activation of pro-neurogenic genes.

Functional and structural chromatin properties are actively regulated in hippocampal NSCs. In fact, and despite being a relatively recent concept in the neuroscience field, the importance of epigenetics on the fine regulation of proliferation, fate specification and differentiation of NSCs is now becoming to be recognised as fundamental for the balanced production of new neuronal and glial cells, necessary for the homeostatic brain function. Therefore, it becomes gradually evident that both extracellular signalling and intracellular epigenetically regulated gene expression programs are dynamically involved in adult neurogenesis. Notably, the intracellular epigenetic program regulating adult neurogenesis is proposed to be quite similar to the epigenetic regulation occurring during development, but is also determined by a myriad of new extrinsic physiological and environmental stimuli [33], that allow the alignment of neurogenesis with the external requests. Even though there is still much to be known, a global picture regarding the epigenetic orchestration of adult neurogenesis commences to emerge (Figure 2).

Epigenetic regulation is implicated in the first stages of adult neurogenesis, by promoting the maintenance of the self-renewal potential of adult NSCs. Molofsky and colleagues [34] highlighted the importance of the trithorax (trxG) and the functionally antagonistic polycomb (PcG) groups of proteins on such regulatory function. The trxG and PcG proteins are chromatin modifier complexes that will, respectively, activate or silence the targeted loci, maintaining such transcriptional state for several cell divisions [35-37]. In particular, the PcG zinc-finger protein Bmi1 has been identified as an important epigenetic regulator, promoting H3K27 methylation and repressing the expression of the cyclin-dependent kinase inhibitor gene p16 (Ink4a); as a consequence the proliferative rate of adult NSCs is maintained [38,39]. In addition, Methyl-CpG binding protein 1 (Mbd1) also appears to be involved in adult NSCs self-renewal in the SGZ. So far, this is the only Mbd protein reported to be specifically involved in hippocampal neurogenesis; Mbd1 deficient mice, although without noticeable developmental deficits, have reduced NSCs proliferation that correlate with cognitive impairments in spatial memory [40]. This protein binds to the promoter region of the gene encoding the mitogen Fibroblast Growth Factor 2 (Fgf2), controlling its expression on adult NSCs of the SGZ. Regulation of the expression of Fgf2 provides the precise control of the timing for the cell to exit the proliferation cycle and to initiate its differentiation [41]. In addition, the MYST family histone acetyltransferase Querkopf (Qkf or Myst4), whose expression was recently described in the adult hippocampus [42], also appears to be involved in the regulation of adult NSCs self-renewal, as NSCs isolated from Qkf mutant mice exhibited a reduced self-renewal capacity [43]. It is important to mention that these epigenetic regulators involved in the adult NSCs maintenance, operate together with several other epigenetic protein regulators such as DNA methyltransferases (DNMTs), histone acetyltransferases (HATs), histone deacetylases (HDACs), and histone methyltransferases (HMTs), that will actively participate in further regulatory steps of the neurogenic process.

When exiting the mitotic phase, neural progenitor cells will eventually become committed to a specific neural cell lineage and start to differentiatiate. Neuronal or glial lineage commitment of NSCs involves a temporal-defined mutual regulation of several gene batteries. Commitment to a neuronal cell-fate, for instance, involves the repression of gliogenic genes; the alternative scenario of glial differentiation, requires the inhibition of genes responsible for neuronal specification. This is achieved through transcriptional and epigenetic regulation, that will integrate also the cell response to the extrinsic environment. In this context, HDACs and HATs are believed to exert an important role in the transduction of physiological signals to the stem cell genome, activating or repressing specific gene programs in NSCs. In general, HDACs catalyze the deacetylation of nucleossomes, that become highly condensed, obstructing the access of transcriptional activation factors to their binding sites and, therefore, resulting in transcriptional repression. In contrast, HATs catalyze the opposite reaction, resulting in global nucleossomal relaxation and, consequently, in increased transcriptional activity [44]. A hint for the importance of these epigenetic modifiers on the neurogenic process, was provided when administrating HDACs inhibitors (HDACis), such as trichostatin A or valproic acid (VPA) on rats. Using such experimental approach, it was shown that HDACis promote neuronal differentiation of adult neural progenitor cells [45]. In addition, at this differentiation stage other proteins, such as trxG proteins, exert their modulatory actions which will ultimately determine cell-fate specification. One interesting example is provided by the trxG family member, Mll1 (mixed-lineage leukaemia 1), which is a H3K4 HMT. Mll1-deficient NSCs retain the capacity to proliferate and fully differentiate into glial lineages, but neuronal differentiation becomes severely compromised. Therefore, Mll1 seems to be required to mediate the transition from a silenced to an active transcriptional state in key loci of postnatal neural precursors necessary for the induction of neurogenesis [46].

Although several other epigenetic regulatory players in cell-fate specification have been identified, it becomes difficult to consider a unifying model through which all epigenetic regulatory actions are coordinated. Nevertheless, a promising candidate for orchestrating these epigenetic events is the DNA binding protein REST (Repressor Element 1 Silencing Transcriprition Factor). REST was first described in 1995, as a repressor of neuronal genes containing a 23 bp conserved sequence, known as RE1 (Repressor Element 1, also named neuron-restrictive silencing factor, or NRSF) [47,48]. This transcription factor coordinates the action of several epigenetic complexes that are required when switching from the undifferentiated stem cell state through the stages of neuronal or glial cell-fate specification [49-51]. After binding to DNA, REST orderly recruits several DNMTs, HMTs, HATs, HDACs, MBDs, co-regulators (CoREST) and cell-cycle proteins, promoting shifts in the overall transcriptional state of specific gene batteries in a cellular context-sensitive manner [49,52]. The recruited epigenetic modulatory proteins, together with specific non-coding RNAs, interact with REST, allowing the precise control of the cellular events that lead to neural progenitors subtype specification [53,54].

Epigenetic regulation is now known to be also implicated in the final maturation of newborn neurons; at this stage it has an important role in promoting the integration of newly-born neurons into the preexisting neural networks. HDACs and HATs appear to be once again decisive for neuronal maturation and early synaptogenesis [55,56]. Using mice deficient on HDAC1 or HDAC2, it was shown that a decreased activity of both enzymes in immature hippocampal neurons triggers excitatory synapse maturation; however, exclusive inhibition of HDAC2 triggers the opposite effect and promotes reduced excitatory synaptic activity [55,57]. Another important epigenetic regulator in the SGZ granule neurons is the methyl-CpG-binding protein 2 (MeCP2). Interestingly, in postmitotic SGZ neurons, the expression of brain-derived neutrophic factor (BDNF), a neurotrophin actively involved in dendritic growth and spine maturation, is associated to reduced DNA methylation and to the release of a chromatin repressive complex comprising MeCP2, in which these cells are highly enriched [58,59]. Furthermore, it has been demonstrated, using knockout mice, that MeCP2 deficiency causes severe deficits in the maturation of newborn neurons in the SGZ, including delayed differentiation and reduced dendritic spine density [60]. In addition, BDNF expression (as well as FGF) can be controlled by other epigenetic regulators such as the DNA-damage inducible protein 45b (Gadd45). This protein is an activity-induced immediate early gene, and its transcription is sensitive to various stimuli that increase neurogenesis by DNA demethylation in mature neurons of neurogenic niches, contributing to the paracrine secretion of neurotrophic factors (BDNF and FGF) that control key processes in adult neurogenesis, including neuronal and dendritic maturation [61,62].

Finally, it is important to note that this epigenetic regulation is not only mediated through cell-intrinsic mechanisms, and that epigenetic mediators are likely stable transducers of extracellular signals (e.g. neuronal activity-dependent or from adjacent glial or endothelial cells) into the regulation of all phases of neurogenesis. The paracrine action of MeCP2 and Gadd45b are amongst the best known examples of this integration of intrinsic and extrinsic signals relevant for neurogenesis regulation [63-65]. Noticeable, these epigenetic regulators exert a complex orchestration of neurogenesis and, therefore, it is plausible that a deregulation of this epigenetic regulatory process is implicated in the neurogenic impairments observed in several neuropsychiatric disorders.

## Role of epigenetic (de)regulation in the ethiopathogenesis of depression: impacts on neurogenesis

### Adult hippocampal neurogenesis on the pathophysiology of depression

Adult hippocampal neurogenesis represents an important, and formerly underestimated, form of neuroplasticity, namely in the hippocampal formation, a brain structure involved in several neuropsychyatric disorders [17,18,66]. Indeed, there is now mounting evidence for the implication of adult hippocampal neurogenesis in the pathophysiology of several neuropsychiatric disorders, a topic extensively reviewed elsewhere [16,19,67]. Perhaps one of the most striking findings in this scientific context was the involvement of adult neurogenesis imbalances in the pathophysiology of major depressive disorder (MDD), as in the action of several antidepressant drugs, thus leading to the substantiation of the so called "neurogenic hypothesis of depression" [68,69]. In fact, several studies have linked reduced neurogenesis to depressive-behaviour and even to the action of several antidepressant drugs [70-73]. Indeed, during the last decade it became obvious the scientific insufficiency of the previously predominant neurochemical-based hypothesis to explain the precipitation of depression, with several authors putting forward alternative underlying mechanisms for the ethiopathogenesis of this disorder [74-76].

Impaired neuronal plasticity is increasingly viewed as central in the ethiopathogenesis of depression. In fact, during the last two decades a significant number of studies in this field revealed cell loss and neuronal atrophy, particularly in brain loci relevant for emotional behaviour control. Several mechanisms were proposed to be responsible for this neuronal atrophy, namely glucocorticoid and glutamate toxicity for both glia and neurons [77], decreased neurotrophic factors expression [78,79], and, more interestingly, decreased neuroplasticity, including dendritic atrophy in the hippocampus in some executive-function brain centres as the prefrontal cortex (PFC) (Bessa et al., 2009b) in animal models for depression. However the most robust link between impaired neuroplasticity and MDD derives from a large number of studies reporting impaired neurogenesis in subjects displaying depressive-like symprtoms [80-82]. Further support to the association of hippocampal neurogenic control and depression, derives from the analysis of the effects of some antidepressants (ADs) in the adult neurogenic process. Counteracting the adverse effects of some of the inducing factors of MDD, ADs bolster neurogenesis in the mammalian hippocampal DG. This pharmacological enhancement of neurogenesis was reported with different classes of ADs, including selective serotonin reuptake inhibitors (SSRIs), monoamine oxidase inhibitors (MAOIs), tricyclic agents and even with putative ADs [70-73,79,83]. Consistent with the results obtained in animal models of depression, ADs also exert this pro-neurogenic effect in non-human primates and humans [84,85].

Lastly, a third link between MDD and hippocampal neurogenesis, is reflected in the functional importance of the adult neurogenic process in some of the behavioural domains commonly affected in depressive patients, such as mood, anxiety and cognition [86-89]. However, the initial proposals claiming that the neurogenic modulatory effects of ADs were responsible for all the behavioural improvements observed after chronic treatment with these drugs is an oversimplification as demonstrated by several studies [70,71,90-93]. In fact, we have demonstrated that the short-term mood-improving actions of antidepressants depended on neuronal remodelling, rather than on neurogenesis [70]. This is not surprising when considering that the pro-neurogenic effects mediated by ADs would not be of neurobiological significance at short-term, since a newborn neuron takes approximately a 5 weeks period to be fully differentiated and integrated in the neuronal circuitry of the adult DG. However, as the majority of studies focus on short-term analysis, one cannot rule out the possibility that the functional contribution of AD-induced neurogenesis will only take place at the long-term. In fact, preliminary data from our lab suggests that despite triggering an immediate pro-neurogenic response, the neurobiological importance of this effect of ADs becomes only significant later on the course of the disease, since the artificial suppression of neurogenesis by the anti-mitotic agent methylazoxymethanol (MAM) significantly compromises behavioural and cognitive long-term recovery, an effect that can be counteracted by ADs treatment (unpublished data).

### Epigenetic (de)regulation of adult neurogenesis as a possible precipitator of depression

**Figure 3:**
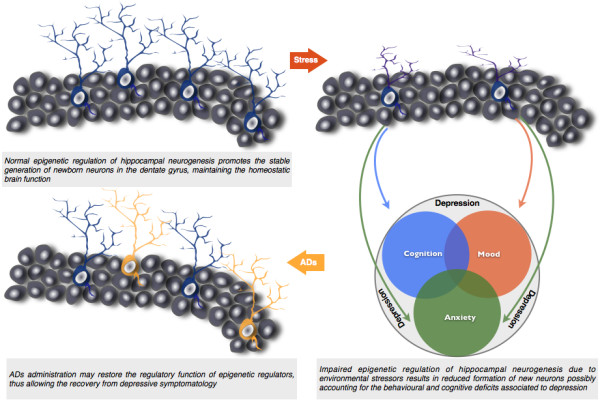
**Role of hippocampal neurogenesis in depression**. Hippocampal neuroplasticity is increasingly viewed as central in the ethiopathogenesis of depression. Chronic exposure to stressors leads to dendritic atrophy on pre-existing granular neurons and compromises the generation of new neurons during adulthood. Data discussed herein strongly suggests that such impairments in the neurogenic process are likely attributable to dysfunctions in the epigenetic regulation of neurogenesis, possibly leading to the multidimensional behavioural deficits associated to depression. Conversely, the question whether the pro-neurogenic action of antidepressants (ADs), which allows to restore normal cognitive function, may be epigenetically mediated remains also to be elucidated.

During the last decade, compelling evidence has emerged for the participation of epigenetic regulatory mechanisms in adult hippocampal neurogenesis. Therefore, it appears reasonable to conceive the hypothesis that dysfunction in epigenetic regulatory mechanisms might mediate the neurogenic imbalances responsible for some neuropsychiatric conditions, such as depression. Although this idea is still controversial, several lines of research suggest that different epigenetic regulatory mechanisms of adult neurogenesis are affected in animal models of depression (Figure 3).

A paradigmatic case supporting this hypothesis is the already mentioned methyl-CpG-binding protein MBD1. In fact, an elucidative work conducted by Allan and colleagues [94], showed that *Mbd1*-deficient mice, besides having decreased NSCs proliferation, as already described by Zhao et al., also exhibit significant deficits in several behavioural dimensions relevant for depression: increased anxious phenotype, detected in both elevated plus maze and light-dark preference tests; behavioural despair, observed in the forced swimming test; and cognitive deficits manifested during the execution of Morris water maze spatial learning tasks [94].

Another example derives from studies focused on MRG15, an active component of HDACs complexes, such as HDAC2 [95,96]. Indeed, *Mrg15*-deficient mice present significant deficits in proliferation of neural progenitors and in their subsequent differentiation [97]. Interestingly, HDAC2 has been identified as a negative regulator of memory, as HDAC2-overexpressing mice presented decreased spine density and synaptic plasticity, that correlates with reduced memory formation [56]. These results confirm the involvement of this histone post-translational modifier protein in controlling both the adult neurogenic process and some of the associated cognitive abilities, also typically affected in stress-related disorders, such as MDD.

Moreover, epigenetic regulators directly involved in post-mitotic neuronal maturation and differentiation, have also been associated with several behavioural and cognitive impairments present in several neuropsychiatric disorders. Work from Adachi et al. [98], for instance, has demonstrated that MeCP2 may interfere in neurological pathways that mediate heightened anxiety. DNMTS, active epigenetic regulators of adult neurogenesis, participating throughout all its phases, are also dynamically involved in blocking memory formation [99,100]. Curiously, DNMT3b, an enzyme responsible for *de novo *DNA methylation, has been reported to have an increased expression in depressive suicide completers. Interestingly, this increase was significantly more pronounced in women, a result that is in accordance with the gender preference of MDD (twice more prevalent in women) [101].

In addition, pharmacological and non-pharmacological treatments of depression, such as ADs and electroconvulsive shock (ECS) therapy, respectively, provide additional endorsement of the hypothesis that the neurogenic precipitation of depression might be, at least partially, epigenetically mediated. In fact, imipramine, a tricyclic agent with a well described pro-neurogenic action [70,79], has behavioural improving actions in a socially defeated mice model, that correlates with downregulation of HDAC5 in the hippocampal region [102]. In contrast, viral-mediated overexpression of HDAC5 counteracts the effects of chronic imipramine treatment in reversing depressive-like behaviour. Interestingly, HDAC5 participates in adult neurogenesis regulation, controlling both maturation and survival of newborn neurons [103]. Interestingly, VPA clinical effectiveness as a mood stabiliser has been correlated to its neurogenic enhancement effect [104,105]. Additional studies showed that VPA, in conjugation with sodium butyrate, when administered alone or in combination with the antidepressant fluoxetine, improves performance in animal models of behavioural despair [106,107]. Considering that VPA is also an HDACi, and that HDAC inhibition is known to drive adult hippocampal neurogenesis [45], these studies highlight the importance of the pharmacological modulation of epigenetic regulators involved in adult neurogenesis to the efficiency of some ADs to ameliorate depressive behaviour. Non-pharmacological treatment of MDD, through ECS therapy, is also known to enhance hippocampal neurogenesis [108]. Interestingly, Gadd45b-deficient mice fail to reveal the ECS-induced increase in adult neurogenesis [62], suggesting that the pro-neurogenic action of ECS therapy, important for reversing depressive-like behaviour, might be epigenetically-mediated by Gadd45b.

Finally, it is worth to mention that this epigenetic regulation is also implicated in the vulnerability to stress, enhancing the susceptibility to stress-related disorders, in which depression is included. The role of such epigenetic mechanisms has recently been highlighted. Indeed, REST4, a splicing variant of the epigenetic orchestrator REST, has been shown to have an increased expression in rats exposed to stress early in life; importantly, these animals display enhanced susceptibility to stress and increased susceptibility to depressive-like behaviour [109]. In addition, KAP1, a crucial component of a repressive chromatin complex, seems also to be involved in stress vulnerability. In fact, mice with deletion of KAP1 in the forebrain exhibit high levels of anxiety-like behaviour and significant stress-induced impairments in some cognitive domains, such as attention and spatial reference memory [110,111]. Therefore, and although not directly involved on neurogenesis regulation, epigenetic-mediated increases in the vulnerability to precipitation factors of depression, such as stress, may lead to an accentuation of the detrimental effects upon the neurogenic process dynamics and its regulatory mechanisms, thus favouring the development of deficits at the behavioural and cognitive levels.

## Conclusions - Towards a neuro-epigenetic hypothesis of depression?

**Figure 4:**
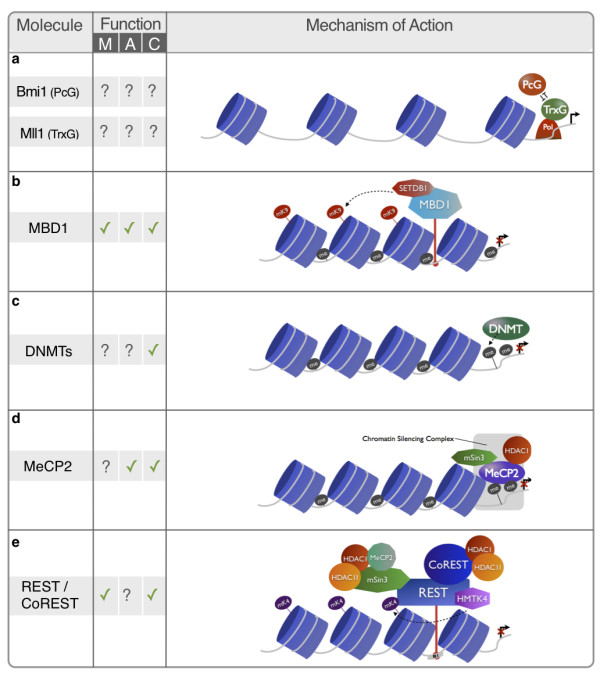
**Epigenetic regulators of neurogenesis on the development of pathological behavioural traits**. Several epigenetic regulators have been implicated in the control of the adult neurogenic process. As such, it is likely that the mechanistic action of these molecules is implicated in the behavioural dimensions commonly affected in depression: Mood (M), Anxiety (A) and Cognition (C). "✓" indicates that such involvement has been described; "?" indicates that the implications of the molecule are unknown or still unclear. **a **PcG and TrxG protein complexes silence or activate, respectively, the transcription of target genes and have been implicated in the control of the neurogenic process; however, repercussions at the behavioural level still remain to be described; **b **MBD1 action is associated with the histone-lysine N-methyltransferase SETDB1, silencing target genes; notably, deficits in this molecule has been associated to deficits in all three behavioural dimension of depression. **c **DNMTs participate in the regulation of a broad range of neurogenesis processes, being its action strongly related to deficits in learning and memory; although some studies suggest that they might be also involved in the transcriptional regulation of pathways associated to mood and anxiety, such correlation needs to be further endorsed; **d **MeCP2 integrates a major chromatin silencing complex comprising several others epigenetic regulators, such as HDAC1, involved in the trancriptional regulation of several genes. Deficits in this molecule have been correlated with cognitive and anxiety deficits, although no deficits in mood have been consistently described; **e **The REST/CoREST chromatin remodeling complex has been proposed as a major orchestrator of the action of several epigenetic regulators, such as HDAC 1 and 2, MeCP2 and the histone methyltransferase K4. Impairments in mood an cognition have been associated with REST and its molecular partners, although no implication have been described relating this molecule to anxiety

The dynamic and environmentally driven modulation of neuroplasticity in the adult brain plays a crucial role in the ethiopathogenesis of depression. As discussed herein, the last two decades provided several lines of evidence supporting the implication of hippocampal neurogenesis in the maintenance of the homeostatic brain function and its importance in the pathophysiology of MDD (Figure 4). However, approaches to this topic have been largely descriptive and the field still lacks an integrative perspective regarding genes and molecular determinants influencing neurogenesis in the installation of such neuropathological conditions; importantly the same holds true for the positive action of ADs in this process.

**Figure 5:**
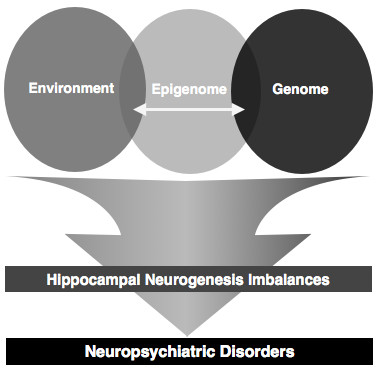
**A neuro-epigenetic hypothesis for the ethiology of neuropsychiatric disease**. Such hypothesis states that Impairments in the environment-genome interplay mediated by epigenetic regulation may account for the imbalances observed at the hippocampal neurogenesis level, thus triggering the onset of the symptomatology typical of such disorders.

Our comprehension of how adult neurogenesis is regulated begins now to be complemented with new insights into the complex epigenetic orchestration of this phenomenon, a regulatory dimension that is relatively new in neuroscience research. Yet, the scattered evidence gathered so far, opens a new dimension for unravelling the mechanistic explanation for the interplay of genes and environment that is central in several neuropsychiatric pathological scenarios. In fact, studies in this area endorse the role of epigenetic mechanisms as transducers of the environmental signals into transcriptional outcomes, strongly suggesting that they may participate in the precipitation of experience-dependent psychiatric disorders, like depression, and simultaneously mediate the pro-neurogenic action of commonly prescribed ADs. Therefore, epigenetic mechanisms begin to emerge as mediators through which environment modulates neurogenesis with long-lasting and stable repercussions in several behavioural and cognitive domains (Figure 5). In this perspective, it becomes plausible that by reverting the pathological effects on epigenetic key regulators, one can counteract the deleterious effects of stress and other precipitators of depression, thus restoring normal neurogenic function. Hence, future research focused on dissecting the epigenetic pathways that modulate the adult neurogenesis process will be decisive to further unravel the neurobiological basis of depression and may pave the way to the development of novel therapies and to the discovery of new therapeutical targets in this pathological context.

## Conflict of interests

The authors declare that they have no competing interests.

## Authors' contributions

AP drafted the manuscript. LP and NS revised the manuscript and coordinated the work. All authors read and approved the final manuscript.

## References

[B1] Doetsch F Caillé I Lim Da García-Verdugo JM Alvarez-Buylla a Subventricular zone astrocytes are neural stem cells in the adult mammalian brain Cell 1999 97 703 716 10.1016/S0092-8674(00)80783-7 10380923

[B2] Gage FH Neurogenesis in the adult brain The Journal of neuroscience: the official journal of the Society for Neuroscience 2002 22 612 613 10.1523/JNEUROSCI.22-03-00612.2002 PMC6758482 11826087

[B3] Silva R Lu J Wu Y Martins L Almeida OF Sousa N Mapping cellular gains and losses in the postnatal dentate gyrus: implications for psychiatric disorders Exp Neurol 2006 200 2 321 331 10.1016/j.expneurol.2006.02.119 16624303

[B4] Brill MS Ninkovic J Winpenny E Hodge RD Ozen I Yang R Lepier A Gascón S Erdelyi F Szabo G Parras C Guillemot F Frotscher M Berninger B Hevner RF Raineteau O Götz M Adult generation of glutamatergic olfactory bulb interneurons Nature neuroscience 2009 12 1524 1533 10.1038/nn.2416 19881504 PMC2787799

[B5] Seri B Garcia-Verdugo JM Collado-Morente L McEwen BS Alvarez-Buylla A Cell types, lineage, and architecture of the germinal zone in the adult dentate gyrus The Journal of comparative neurology 2004 478 359 378 10.1002/cne.20288 15384070

[B6] Chumley MJ Catchpole T Silvany RE Kernie SG Henkemeyer M EphB Receptors Regulate Stem/Progenitor Cell Proliferation, Migration, and Polarity during Hippocampal Neurogenesis Journal of Neuroscience 2007 27 13481 13490 10.1523/JNEUROSCI.4158-07.2007 18057206 PMC6673089

[B7] Wu W Wong K Chen J Jiang Z Dupuis S Wu JY Rao Y Directional guidance of neuronal migration in the olfactory system by the protein Slit Nature 1999 400 331 10.1038/22477 10432110 PMC2041931

[B8] Luzzati F De Marchis S Fasolo A Peretto P Neurogenesis in the caudate nucleus of the adult rabbit Journal of Neuroscience 2006 26 609 10.1523/JNEUROSCI.4371-05.2006 16407559 PMC6674396

[B9] Kodama M Fujioka T Duman RS Chronic olanzapine or fluoxetine administration increases cell proliferation in hippocampus and prefrontal cortex of adult rat Biological psychiatry 2004 56 570 580 10.1016/j.biopsych.2004.07.008 15476686

[B10] Ohira K Furuta T Hioki H Nakamura KC Kuramoto E Tanaka Y Funatsu N Shimizu K Oishi T Hayashi M Ischemia-induced neurogenesis of neocortical layer 1 progenitor cells Nature Neuroscience 2009 13 173 180 20037576 10.1038/nn.2473

[B11] Gonçalves L Silva R Pinto-Ribeiro F Pego JM Bessa JM Pertovaara A Sousa N Almeida A Neuropathic pain is associated with depressive behaviour and induces neuroplasticity in the amygdala of the rat Exp Neurol 2008 213 1 48 56 10.1016/j.expneurol.2008.04.043 18599044

[B12] Fowler CD Liu Y Ouimet C Wang Z The effects of social environment on adult neurogenesis in the female prairie vole Journal of neurobiology 2002 51 115 128 10.1002/neu.10042 11932953

[B13] Kokoeva MV Yin H Flier JS Neurogenesis in the hypothalamus of adult mice: potential role in energy balance Science (New York, NY) 2005 310 679 683 10.1126/science.1115360 16254185

[B14] Chmielnicki E Benraiss A Economides AN Goldman Sa Adenovirally expressed noggin and brain-derived neurotrophic factor cooperate to induce new medium spiny neurons from resident progenitor cells in the adult striatal ventricular zone The Journal of neuroscience: the official journal of the Society for Neuroscience 2004 24 2133 2142 10.1523/JNEUROSCI.1554-03.2004 PMC6730416 14999064

[B15] Ehninger D Kempermann G Regional Effects of Wheel Running and Environmental Enrichment on Cell Genesis and Microglia Proliferation in the Adult Murine Neocortex Cerebral Cortex 2003 845 851 10.1093/cercor/13.8.845 12853371

[B16] Eisch AJ Cameron Ha Encinas JM Meltzer La Ming G-L Overstreet-Wadiche LS Adult neurogenesis, mental health, and mental illness: hope or hype? The Journal of neuroscience: the official journal of the Society for Neuroscience 2008 28 11785 11791 10.1523/JNEUROSCI.3798-08.2008 PMC2793333 19005040

[B17] Kobayashi K Targeting the hippocampal mossy fiber synapse for the treatment of psychiatric disorders Molecular neurobiology 2009 39 24 36 10.1007/s12035-008-8049-5 19130314

[B18] Sapolsky RM Glucocorticoids and hippocampal atrophy in neuropsychiatric disorders Archives of general psychiatry 2000 57 925 10.1001/archpsyc.57.10.925 11015810

[B19] Balu DT Lucki I Adult hippocampal neurogenesis: regulation, functional implications, and contribution to disease pathology Neuroscience and biobehavioral reviews 2009 33 232 252 10.1016/j.neubiorev.2008.08.007 18786562 PMC2671071

[B20] Kempermann G Jessberger S Steiner B Kronenberg G Milestones of neuronal development in the adult hippocampus Trends in neurosciences 2004 27 447 452 10.1016/j.tins.2004.05.013 15271491

[B21] Steiner B Klempin F Wang L Kott M Kettenmann H Kempermann G Type-2 cells as link between glial and neuronal lineage in adult hippocampal neurogenesis Glia 2006 54 805 814 10.1002/glia.20407 16958090

[B22] Seri B Garcia-Verdugo JM McEwen BS Alvarez-Buylla A Astrocytes give rise to new neurons in the adult mammalian hippocampus Journal of Neuroscience 2001 21 7153 11549726 10.1523/JNEUROSCI.21-18-07153.2001 PMC6762987

[B23] Filippov V Subpopulation of nestin-expressing progenitor cells in the adult murine hippocampus shows electrophysiological and morphological characteristics of astrocytes Molecular and Cellular Neuroscience 2003 23 373 382 10.1016/S1044-7431(03)00060-5 12837622

[B24] Rakic P Elusive radial glial cells: historical and evolutionary perspective Glia 2003 43 19 32 10.1002/glia.10244 12761862

[B25] Encinas JM Vaahtokari A Enikolopov G Fluoxetine targets early progenitor cells in the adult brain Proceedings of the National Academy of Sciences of the United States of America 2006 103 8233 8238 10.1073/pnas.0601992103 16702546 PMC1461404

[B26] Pleasure SJ Collins aE Lowenstein DH Unique expression patterns of cell fate molecules delineate sequential stages of dentate gyrus development The Journal of neuroscience: the official journal of the Society for Neuroscience 2000 20 6095 6105 10.1523/JNEUROSCI.20-16-06095.2000 PMC6772596 10934259

[B27] Espósito MS Piatti VC Laplagne Da Morgenstern Na Ferrari CC Pitossi FJ Schinder AF Neuronal differentiation in the adult hippocampus recapitulates embryonic development The Journal of neuroscience: the official journal of the Society for Neuroscience 2005 25 10074 10086 10.1523/JNEUROSCI.3114-05.2005 PMC6725804 16267214

[B28] Zhao C Teng EM Summers RG Ming G-L Gage FH Distinct morphological stages of dentate granule neuron maturation in the adult mouse hippocampus The Journal of neuroscience: the official journal of the Society for Neuroscience 2006 26 3 11 10.1523/JNEUROSCI.3648-05.2006 PMC6674324 16399667

[B29] Ambrogini P Lattanzi D Ciuffoli S Agostini D Bertini L Stocchi V Santi S Cuppini R Morpho-functional characterization of neuronal cells at different stages of maturation in granule cell layer of adult rat dentate gyrus Brain Res 2004 1017 1-2 21 31 10.1016/j.brainres.2004.05.039 15261095

[B30] van Praag H Schinder AF Christie BR Toni N Palmer TD Gage FH Functional neurogenesis in the adult hippocampus Nature 2002 415 6875 10q 1034 10.1038/4151030a PMC9284568 11875571

[B31] Biebl M Cooper CM Winkler J Kuhn HG Analysis of neurogenesis and programmed cell death reveals a self-renewing capacity in the adult rat brain Neurosci Lett 2000 291 1 17 20 10.1016/S0304-3940(00)01368-9 10962143

[B32] Kuhn HG Biebl M Wilhelm D Li M Friedlander RM Winkler J Increased generation of granule cells in adult Bcl-2-overexpressing mice: a role for cell death during continued hippocampal neurogenesis Eur J Neurosci 2005 22 8 1907 1915 10.1111/j.1460-9568.2005.04377.x 16262630

[B33] Ninkovic J Götz M Signaling in adult neurogenesis: from stem cell niche to neuronal networks Current opinion in neurobiology 2007 17 338 344 10.1016/j.conb.2007.04.006 17475475

[B34] Molofsky AV Pardal R Iwashita T Park IK Clarke MF Morrison SJ Bmi-1 dependence distinguishes neural stem cell self-renewal from progenitor proliferation Nature 2003 425 962 967 10.1038/nature02060 14574365 PMC2614897

[B35] Ng RK Gurdon JB Epigenetic inheritance of cell differentiation status Cell Cycle 2008 7 1173 1177 10.4161/cc.7.9.5791 18418041

[B36] Ringrose L Paro R Polycomb/Trithorax response elements and epigenetic memory of cell identity Development (Cambridge, England) 2007 134 223 232 10.1242/dev.02723 17185323

[B37] Schuettengruber B Chourrout D Vervoort M Leblanc B Cavalli G Genome regulation by polycomb and trithorax proteins Cell 2007 128 735 745 10.1016/j.cell.2007.02.009 17320510

[B38] Fasano Ca Dimos JT Ivanova NB Lowry N Lemischka IR Temple S shRNA knockdown of Bmi-1 reveals a critical role for p21-Rb pathway in NSC self-renewal during development Cell stem cell 2007 1 87 99 10.1016/j.stem.2007.04.001 18371338

[B39] Fasano Ca Phoenix TN Kokovay E Lowry N Elkabetz Y Dimos JT Lemischka IR Studer L Temple S Bmi-1 cooperates with Foxg1 to maintain neural stem cell self-renewal in the forebrain Genes & development 2009 23 561 574 10.1101/gad.1743709 19270157 PMC2658524

[B40] Zhao X Ueba T Christie BR Barkho B McConnell MJ Nakashima K Lein ES Eadie BD Willhoite AR Muotri AR Summers RG Chun J Lee K-F Gage FH Mice lacking methyl-CpG binding protein 1 have deficits in adult neurogenesis and hippocampal function Proceedings of the National Academy of Sciences of the United States of America 2003 100 6777 6782 10.1073/pnas.1131928100 12748381 PMC164523

[B41] Li X Barkho BZ Luo Y Smrt RD Santistevan NJ Liu C Kuwabara T Gage FH Zhao X Epigenetic regulation of the stem cell mitogen Fgf-2 by Mbd1 in adult neural stem/progenitor cells The Journal of biological chemistry 2008 283 27644 27652 10.1074/jbc.M804899200 18689796 PMC2562066

[B42] Maisel M Herr A Milosevic J Hermann A Habisch H-J Schwarz S Kirsch M Antoniadis G Brenner R Hallmeyer-Elgner S Lerche H Schwarz J Storch A Transcription profiling of adult and fetal human neuroprogenitors identifies divergent paths to maintain the neuroprogenitor cell state Stem cells (Dayton, Ohio) 2007 25 1231 1240 10.1634/stemcells.2006-0617 17218394

[B43] Merson TD Dixon MP Collin C Rietze RL Bartlett PF Thomas T Voss AK The transcriptional coactivator Querkopf controls adult neurogenesis The Journal of neuroscience: the official journal of the Society for Neuroscience 2006 26 11359 11370 10.1523/JNEUROSCI.2247-06.2006 PMC6674553 17079664

[B44] Mehler MF Epigenetic principles and mechanisms underlying nervous system functions in health and disease Progress in neurobiology 2008 86 305 341 10.1016/j.pneurobio.2008.10.001 18940229 PMC2636693

[B45] Hsieh J Nakashima K Kuwabara T Mejia E Gage FH Histone deacetylase inhibition-mediated neuronal differentiation of multipotent adult neural progenitor cells Proceedings of the National Academy of Sciences of the United States of America 2004 101 16659 16664 10.1073/pnas.0407643101 15537713 PMC527137

[B46] Lim Da Huang Y-C Swigut T Mirick AL Garcia-Verdugo JM Wysocka J Ernst P Alvarez-Buylla A Chromatin remodelling factor Mll1 is essential for neurogenesis from postnatal neural stem cells Nature 2009 458 529 533 10.1038/nature07726 19212323 PMC3800116

[B47] Chong Ja Tapia-Ramírez J Kim S Toledo-Aral JJ Zheng Y Boutros MC Altshuller YM Frohman Ma Kraner SD Mandel G REST: a mammalian silencer protein that restricts sodium channel gene expression to neurons Cell 1995 80 949 957 10.1016/0092-8674(95)90298-8 7697725

[B48] Schoenherr CJ Anderson DJ Silencing is golden: negative regulation in the control of neuronal gene transcription Current opinion in neurobiology 1995 5 566 571 10.1016/0959-4388(95)80060-3 8580707

[B49] Ooi L Wood IC Chromatin crosstalk in development and disease: lessons from REST Nature reviews Genetics 2007 8 544 554 10.1038/nrg2100 17572692

[B50] Otto SJ McCorkle SR Hover J Conaco C Han J-J Impey S Yochum GS Dunn JJ Goodman RH Mandel G A new binding motif for the transcriptional repressor REST uncovers large gene networks devoted to neuronal functions The Journal of neuroscience: the official journal of the Society for Neuroscience 2007 27 6729 6739 10.1523/JNEUROSCI.0091-07.2007 PMC6672685 17581960

[B51] Singh SK Kagalwala MN Parker-Thornburg J Adams H Majumder S REST maintains self-renewal and pluripotency of embryonic stem cells Nature 2008 453 223 227 10.1038/nature06863 18362916 PMC2830094

[B52] Ballas N Mandel G The many faces of REST oversee epigenetic programming of neuronal genes Current opinion in neurobiology 2005 15 500 506 10.1016/j.conb.2005.08.015 16150588

[B53] Visvanathan J Lee S Lee B Lee JW Lee S-K The microRNA miR-124 antagonizes the anti-neural REST/SCP1 pathway during embryonic CNS development Genes & development 2007 21 744 749 10.1101/gad.1519107 17403776 PMC1838526

[B54] Wu J Xie X Comparative sequence analysis reveals an intricate network among REST, CREB and miRNA in mediating neuronal gene expression Genome biology 2006 7 R85 10.1186/gb-2006-7-9-r85 17002790 PMC1794552

[B55] Akhtar MW Raingo J Nelson ED Montgomery RL Olson EN Kavalali ET Monteggia LM Histone deacetylases 1 and 2 form a developmental switch that controls excitatory synapse maturation and function J Neurosci 2009 29 25 8288 8297 10.1523/JNEUROSCI.0097-09.2009 19553468 PMC2895817

[B56] Guan J-S Haggarty SJ Giacometti E Dannenberg J-H Joseph N Gao J Nieland TJF Zhou Y Wang X Mazitschek R Bradner JE DePinho Ra Jaenisch R Tsai L-H HDAC2 negatively regulates memory formation and synaptic plasticity Nature 2009 459 55 60 10.1038/nature07925 19424149 PMC3498958

[B57] Jawerka M Colak D Dimou L Spiller C Lagger S Montgomery RL Olson EN Wurst W Gottlicher M Gotz M The specific role of histone deacetylase 2 in adult neurogenesis Neuron Glia Biol 2010 6 2 93 107 10.1017/S1740925X10000049 20388229

[B58] Martinowich K Hattori D Wu H Fouse S He F Hu Y Fan G Sun YE DNA methylation-related chromatin remodeling in activity-dependent BDNF gene regulation Science (New York, NY) 2003 302 890 893 10.1126/science.1090842 14593184

[B59] Zhou Z Hong EJ Cohen S Zhao W-N Ho H-YH Schmidt L Chen WG Lin Y Savner E Griffith EC Hu L Steen JaJ Weitz CJ Greenberg ME Brain-specific phosphorylation of MeCP2 regulates activity-dependent Bdnf transcription, dendritic growth, and spine maturation Neuron 2006 52 255 269 10.1016/j.neuron.2006.09.037 17046689 PMC3962021

[B60] Smrt RD Eaves-Egenes J Barkho BZ Santistevan NJ Zhao C Aimone JB Gage FH Zhao X Mecp2 deficiency leads to delayed maturation and altered gene expression in hippocampal neurons Neurobiol Dis 2007 27 1 77 89 10.1016/j.nbd.2007.04.005 17532643 PMC2789309

[B61] Ma DK Guo JU Ming G Song H DNA excision repair proteins and Gadd45 as molecular players for active DNA demethylation Cell cycle (Georgetown, Tex) 2009 8 1526 10.4161/cc.8.10.8500 PMC2738863 19377292

[B62] Ma DK Jang M-h Guo JU Kitabatake Y Chang M-L Pow-Anpongkul N Flavell RA Lu B Ming G-l Song H Neuronal activity-induced Gadd45b promotes epigenetic DNA demethylation and adult neurogenesis Science (New York, NY) 2009 323 1074 1077 10.1126/science.1166859 PMC2726986 19119186

[B63] Mu Y Lee S Signaling in adult neurogenesis Current Opinion in Neurobiology 2010 20 416 423 10.1016/j.conb.2010.04.010 20471243 PMC2942965

[B64] Shen Q Goderie SK Jin L Karanth N Sun Y Abramova N Vincent P Pumiglia K Temple S Endothelial cells stimulate self-renewal and expand neurogenesis of neural stem cells Science (New York, NY) 2004 304 1338 1340 10.1126/science.1095505 15060285

[B65] Ma DK Ming G-L Song H Glial influences on neural stem cell development: cellular niches for adult neurogenesis Current opinion in neurobiology 2005 15 514 520 10.1016/j.conb.2005.08.003 16144763

[B66] Hsieh J Eisch AJ Epigenetics, hippocampal neurogenesis, and neuropsychiatric disorders: unraveling the genome to understand the mind Neurobiol Dis 2010 39 1 73 84 10.1016/j.nbd.2010.01.008 20114075 PMC2874108

[B67] DeCarolis Na Eisch AJ Hippocampal neurogenesis as a target for the treatment of mental illness: a critical evaluation Neuropharmacology 2010 58 884 893 10.1016/j.neuropharm.2009.12.013 20060007 PMC2839019

[B68] Duman RS Role of neurotrophic factors in the etiology and treatment of mood disorders Neuromolecular Med 2004 5 1 11 25 10.1385/NMM:5:1:011 15001809

[B69] Warner-Schmidt JL Duman RS Hippocampal neurogenesis: opposing effects of stress and antidepressant treatment Hippocampus 2006 16 3 239 249 10.1002/hipo.20156 16425236

[B70] Bessa JM Palha JA Almeida OFX Ferreira D Sousa N Melo I Marques F Cerqueira JJ The mood-improving actions of antidepressants do not depend on neurogenesis but are associated with neuronal remodeling Molecular psychiatry 2009 14 764 773 739 10.1038/mp.2008.119 18982002

[B71] David DJ Samuels BA Rainer Q Wang J-W Marsteller D Mendez I Drew M Craig Da Guiard BP Guilloux J-P Artymyshyn RP Gardier AM Gerald C Antonijevic Ia Leonardo ED Hen R Neurogenesis-dependent and -independent effects of fluoxetine in an animal model of anxiety/depression Neuron 2009 62 479 493 10.1016/j.neuron.2009.04.017 19477151 PMC2759281

[B72] Malberg JE Eisch AJ Nestler EJ Duman RS Chronic antidepressant treatment increases neurogenesis in adult rat hippocampus Journal of Neuroscience 2000 20 9104 11124987 10.1523/JNEUROSCI.20-24-09104.2000 PMC6773038

[B73] Santarelli L Saxe M Gross C Surget A Battaglia F Dulawa S Weisstaub N Lee J Duman R Arancio O Belzung C Hen R Requirement of hippocampal neurogenesis for the behavioral effects of antidepressants Science (New York, NY) 2003 301 805 809 10.1126/science.1083328 12907793

[B74] Castrén E Is mood chemistry? Nature reviews Neuroscience 2005 6 241 246 15738959 10.1038/nrn1629

[B75] Pittenger C Duman RS Stress, depression, and neuroplasticity: a convergence of mechanisms Neuropsychopharmacology: official publication of the American College of Neuropsychopharmacology 2008 33 88 109 10.1038/sj.npp.1301574 17851537

[B76] Sousa N Madeira MD Paula-Barbosa MM Effects of corticosterone treatment and rehabilitation on the hippocampal formation of neonatal and adult rats An unbiased stereological study. Brain research 1998 794 199 210 10.1016/s0006-8993(98)00218-2 9622630

[B77] Abraham I Juhasz G Kekesi KA Kovacs KJ Corticosterone peak is responsible for stress-induced elevation of glutamate in the hippocampus Stress 1998 2 3 171 181 10.3109/10253899809167281 9787265

[B78] Lee J Duan W Mattson MP Evidence that brain-derived neurotrophic factor is required for basal neurogenesis and mediates, in part, the enhancement of neurogenesis by dietary restriction in the hippocampus of adult mice Journal of neurochemistry 2002 82 1367 1375 10.1046/j.1471-4159.2002.01085.x 12354284

[B79] Sairanen M Lucas G Ernfors P Castrén M Castrén E Brain-derived neurotrophic factor and antidepressant drugs have different but coordinated effects on neuronal turnover, proliferation, and survival in the adult dentate gyrus The Journal of neuroscience: the official journal of the Society for Neuroscience 2005 25 1089 1094 10.1523/JNEUROSCI.3741-04.2005 PMC6725966 15689544

[B80] Alonso R Griebel G Pavone G Stemmelin J Le Fur G Soubrié P Blockade of CRF1 or V1B receptors reverses stress-induced suppression of neurogenesis in a mouse model of depression Molecular Psychiatry 2004 9 224 224 10.1038/sj.mp.4001484 14699428

[B81] Coe C Prenatal stress diminishes neurogenesis in the dentate gyrus of juvenile Rhesus monkeys Biological Psychiatry 2003 54 1025 1034 10.1016/S0006-3223(03)00698-X 14625144

[B82] Sahay A Hen R Adult hippocampal neurogenesis in depression Nature neuroscience 2007 10 1110 1115 10.1038/nn1969 17726477

[B83] Banasr M Valentine GW Li X-Y Gourley SL Taylor JR Duman RS Chronic unpredictable stress decreases cell proliferation in the cerebral cortex of the adult rat Biological psychiatry 2007 62 496 504 10.1016/j.biopsych.2007.02.006 17585885

[B84] Boldrini M Underwood MD Hen R Rosoklija GB Dwork AJ Mann JJ Arango V Antidepressants increase neural progenitor cells in the human hippocampus Neuropsychopharmacology: official publication of the American College of Neuropsychopharmacology 2009 34 2376 2389 10.1038/npp.2009.75 PMC2743790 19606083

[B85] Perera TD Coplan JD Lisanby SH Lipira CM Arif M Carpio C Spitzer G Santarelli L Scharf B Hen R Rosoklija G Sackeim Ha Dwork AJ Antidepressant-induced neurogenesis in the hippocampus of adult nonhuman primates The Journal of neuroscience: the official journal of the Society for Neuroscience 2007 27 4894 4901 10.1523/JNEUROSCI.0237-07.2007 PMC6672102 17475797

[B86] Bessa JM Mesquita AR Oliveira M Pêgo JM Cerqueira JJ Palha Ja Almeida OFX Sousa N A trans-dimensional approach to the behavioral aspects of depression Frontiers in behavioral neuroscience 2009 3 1 19194528 10.3389/neuro.08.001.2009 PMC2634526

[B87] Clelland CD Choi M Romberg C Clemenson GD Fragniere a Tyers P Jessberger S Saksida LM Barker Ra Gage FH Bussey TJ A functional role for adult hippocampal neurogenesis in spatial pattern separation Science (New York, NY) 2009 325 210 213 10.1126/science.1173215 PMC2997634 19590004

[B88] Dupret D Revest J-M Koehl M Ichas F De Giorgi F Costet P Abrous DN Piazza PV Spatial relational memory requires hippocampal adult neurogenesis PloS one 2008 3 e1959 10.1371/journal.pone.0001959 18509506 PMC2396793

[B89] Kee N Teixeira CM Wang AH Frankland PW Preferential incorporation of adult-generated granule cells into spatial memory networks in the dentate gyrus Nature neuroscience 2007 10 355 362 10.1038/nn1847 17277773

[B90] Holick Ka Lee DC Hen R Dulawa SC Behavioral effects of chronic fluoxetine in BALB/cJ mice do not require adult hippocampal neurogenesis or the serotonin 1A receptor Neuropsychopharmacology: official publication of the American College of Neuropsychopharmacology 2008 33 406 417 10.1038/sj.npp.1301399 17429410

[B91] Jayatissa MN Henningsen K West MJ Wiborg O Decreased cell proliferation in the dentate gyrus does not associate with development of anhedonic-like symptoms in rats Brain research 2009 1290 133 141 19595674 10.1016/j.brainres.2009.07.001

[B92] Sapolsky RM Is impaired neurogenesis relevant to the affective symptoms of depression? Biological psychiatry 2004 56 137 139 10.1016/j.biopsych.2004.04.012 15271580

[B93] Singer BH Jutkiewicz EM Fuller CL Lichtenwalner RJ Zhang H Velander AJ Li X Gnegy ME Burant CF Parent JM Conditional ablation and recovery of forebrain neurogenesis in the mouse The Journal of comparative neurology 2009 514 567 582 10.1002/cne.22052 19363795 PMC2739050

[B94] Allan AM Liang X Luo Y Pak C Li X Szulwach KE Chen D Jin P Zhao X The loss of methyl-CpG binding protein 1 leads to autism-like behavioral deficits Access 2008 17 2047 2057 10.1093/hmg/ddn102 PMC2700628 18385101

[B95] Bhaumik SR Smith E Shilatifard A Covalent modifications of histones during development and disease pathogenesis Nature structural & molecular biology 2007 14 1008 1016 10.1038/nsmb1337 17984963

[B96] Garcia SN Pereira-Smith O MRGing chromatin dynamics and cellular senescence Cell biochemistry and biophysics 2008 50 133 141 10.1007/s12013-008-9006-7 18231726

[B97] Chen M Takano-Maruyama M Pereira-Smith OM Gaufo GO Tominaga K MRG15, a component of HAT and HDAC complexes, is essential for proliferation and differentiation of neural precursor cells Journal of neuroscience research 2009 87 1522 1531 10.1002/jnr.21976 19115414 PMC2913448

[B98] Adachi M Autry AE Covington HE Monteggia LM MeCP2-mediated transcription repression in the basolateral amygdala may underlie heightened anxiety in a mouse model of Rett syndrome The Journal of neuroscience: the official journal of the Society for Neuroscience 2009 29 4218 4227 10.1523/JNEUROSCI.4225-08.2009 PMC3005250 19339616

[B99] Miller Ca Campbell SL Sweatt JD DNA methylation and histone acetylation work in concert to regulate memory formation and synaptic plasticity Neurobiology of learning and memory 2008 89 599 603 10.1016/j.nlm.2007.07.016 17881251 PMC2430891

[B100] Miller Ca Sweatt JD Covalent modification of DNA regulates memory formation Neuron 2007 53 857 869 10.1016/j.neuron.2007.02.022 17359920

[B101] Poulter MO Du L Weaver ICG Palkovits M Faludi G Merali Z Szyf M Anisman H GABA(A) receptor promoter hypermethylation in suicide brain: implications for the involvement of epigenetic processes Biological psychiatry 2008 64 645 652 10.1016/j.biopsych.2008.05.028 18639864

[B102] Tsankova NM Berton O Renthal W Kumar A Neve RL Nestler EJ Sustained hippocampal chromatin regulation in a mouse model of depression and antidepressant action Nature neuroscience 2006 9 519 525 10.1038/nn1659 16501568

[B103] Schneider JW Gao Z Li S Farooqi M Tang T-S Bezprozvanny I Frantz DE Hsieh J Small-molecule activation of neuronal cell fate Nature chemical biology 2008 4 408 410 18552832 10.1038/nchembio.95

[B104] Manji HK Moore GJ Chen G Clinical and preclinical evidence for the neurotrophic effects of mood stabilizers: implications for the pathophysiology and treatment of manic-depressive illness Biological psychiatry 2000 48 740 754 10.1016/S0006-3223(00)00979-3 11063971

[B105] Yu IT Park J-Y Kim SH Lee J-S Kim Y-S Son H Valproic acid promotes neuronal differentiation by induction of proneural factors in association with H4 acetylation Neuropharmacology 2009 56 473 480 10.1016/j.neuropharm.2008.09.019 19007798

[B106] Semba J Kuroda Y Takahashi R Potential antidepressant properties of subchronic GABA transaminase inhibitors in the forced swimming test in mice Neuropsychobiology 1989 21 3 152 156 10.1159/000118569 2559361

[B107] Schroeder Fa Lin CL Crusio WE Akbarian S Antidepressant-like effects of the histone deacetylase inhibitor, sodium butyrate, in the mouse Biological psychiatry 2007 62 55 64 10.1016/j.biopsych.2006.06.036 16945350

[B108] Madsen TM Treschow a Bengzon J Bolwig TG Lindvall O Tingström a Increased neurogenesis in a model of electroconvulsive therapy Biological psychiatry 2000 47 1043 1049 10.1016/S0006-3223(00)00228-6 10862803

[B109] Uchida S Hara K Kobayashi A Funato H Hobara T Otsuki K Yamagata H McEwen BS Watanabe Y Early life stress enhances behavioral vulnerability to stress through the activation of REST4-mediated gene transcription in the medial prefrontal cortex of rodents J Neurosci 2010 30 45 15007 15018 10.1523/JNEUROSCI.1436-10.2010 21068306 PMC6633839

[B110] Bredy TW Sun YE Kobor MS How the epigenome contributes to the development of psychiatric disorders Developmental psychobiology 2010 52 331 342 10.1002/dev.20424 20127889 PMC2946415

[B111] Jakobsson J Cordero MI Bisaz R Groner AC Busskamp V Bensadoun J-C Cammas F Losson R Mansuy IM Sandi C Trono D KAP1-mediated epigenetic repression in the forebrain modulates behavioral vulnerability to stress Neuron 2008 60 818 831 10.1016/j.neuron.2008.09.036 19081377

